# Systematic Characterization of Electronic Metal–Support Interactions in Ceria-Supported Pt Particles

**DOI:** 10.1021/acs.jpcc.3c03383

**Published:** 2023-08-30

**Authors:** Pablo Castro-Latorre, Konstantin M. Neyman, Albert Bruix

**Affiliations:** † Departament de Ciència de Materials i Química Física, Institut de Quimica Teòrica i Computacional (IQTCUB), Universitat de Barcelona, 08028 Barcelona, Spain; ‡ ICREA (Institució Catalana de Recerca i Estudis Avançats), 08010 Barcelona, Spain

## Abstract

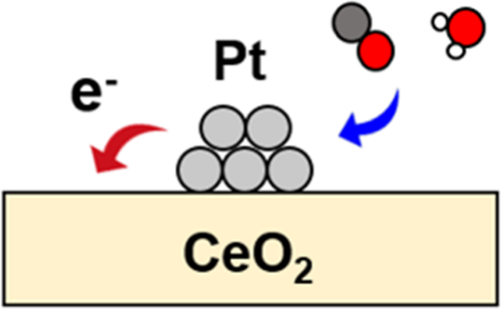

Electronic metal–support interactions affect the chemical and catalytic properties of metal particles supported on reducible metal oxides, but their characterization is challenging due to the complexity of the electronic structure of these systems. These interactions often involve different states with varying numbers and positions of strongly correlated *d* or *f* electrons and the corresponding polarons. In this work, we present an approach to characterize electronic metal–support interactions by means of computationally efficient density functional calculations within the projector augmented wave method. We describe Ce^3+^ cations with potentials that include a Ce4*f* electron in the frozen core, overcoming prevalent convergence and 4*f* electron localization issues. We systematically explore the stability and chemical properties of different electronic states for a Pt_8_/CeO_2_(111) model system, revealing the predominant effect of electronic metal–support interactions on Pt atoms located directly at the metal–oxide interface. Adsorption energies and the reactivity of these interface Pt atoms vary significantly upon donation of electrons to the oxide support, pointing to a strategy to selectively activate interfacial sites of metal particles supported on reducible metal oxides.

## 1 Introduction

Cerium dioxide (CeO_2_, ceria) is a technologically relevant material due to its catalytic applications in fuel cells, the three-way catalysis, or the water gas shift reaction, among other reactions.^1−6^ One of the key properties of CeO_2_ is its reducibility, involving the reduction of Ce^4+^ centers to Ce^3+^ upon formation of oxygen vacancies^7−12^ or electron transfer processes.^13−16^ The latter typically involve supported metal particles as electron donors, resulting in complex distortions of the electronic structure of both metal and ceria supports.^15,17−23^ The effects of these interactions on chemical properties were first identified for ceria-supported Pt particles in catalysts for the water gas shift reaction^24^ and later coined by Campbell as electronic metal–support interactions (EMSI) in a news and views article.^25^ EMSI are sometimes referred to as strong metal–support interactions (SMSI), which were traditionally considered as pronounced structural (instead of electronic) distortions of the metal–oxide interface detrimental to catalyst activity^26^ but are now used more generally to describe any significant effect of the metal–support interface on reactivity.^27,28^

EMSI are exhibited by various combinations of supports and metals,^28−31^ among which ceria-supported transition metals are frequently explored.^32−35^ Transition-metal particles often become somewhat oxidized upon contact with a reducible support such as ceria, which can affect adsorption energies and dissociation barriers of key steps within a reaction mechanism. EMSI are also markedly size-dependent, with large particles, clusters, and single atoms exhibiting different propensity for oxidation.^19,36−38^ Despite recent work elucidating some aspects of EMSI, the stability of different electronic states and the effect of the oxidation state of supported metal particles on their chemical reactivity and catalytic properties are generally not well understood.

Theoretical calculations based on density functional theory (DFT) have led the characterization of the charge distribution and redox behavior of these systems. However, these calculations are challenging due to the strongly correlated nature of *d* and *f* electrons in some reducible oxides. A correction in the form of a Hubbard *U* or a fraction of the exact exchange is often applied to partially counteract the self-interaction error and the resulting spurious electron delocalization. These parametrized approaches are, however, not quantitatively reliable because calculated results can notably depend on the magnitude of the applied correction.^37^ One is therefore often satisfied with describing general qualitative trends that do not disappear when varying the *U* value or the fraction of exact exchange used in the calculation.

Even when employing DFT+*U* or hybrid functionals, another important obstacle to characterizing EMSI is a challenge to carry out calculations that converge to the desired electronic state for a given system. The number and position of 4*f* electrons in Ce atoms of a ceria-based system is hard to control. In addition, many periodic codes such as VASP^39−41^ often do not converge to the most stable electronic state, and in such cases, sampling of various states is required. To overcome this, some authors have developed methods within VASP^42,43^ or other codes^44,45^ to control the orbital occupancy matrix and converge to the desired state. In this work, we propose another approach to converge to the desired electronic states by considering the *f* electrons of ceria as fixed core electrons of the potential used within the projector augmented wave method.^39,41,46^ We demonstrate that using such pseudopotential allows characterizing the electronic structure and chemical properties of ceria-supported Pt particles without the need to consider spin polarization, which makes the systematic characterization of EMSI for these systems computationally efficient (low cost) and straightforward (easy to converge). The systematic characterization with this approach of a previously studied Pt_8_/CeO_2_(111) model system reveals that the effects of EMSI are strongly site-dependent and that transfer of electrons from the platinum particle to the ceria support can even invert the preference for adsorption between interface and noninterface Pt sites.

## 2 Computational Methods and Models

### 2.1 Computational Methods

Density functional theory calculations were performed using periodic models as implemented in the VASP software package.^39−41^ Valence states were described by plane wave basis sets with an energy cutoff of 415 eV, and the projector augmented wave (PAW) method of Blöchl^46^ was used to account for the interaction between fixed core and explicitly described valence electrons. The PAW potentials used describe 2s and 2p electrons explicitly for O and 5*d* and 6*s* electrons for Pt. For Ce atoms, we used two different PAW potentials. The most used VASP potential for Ce describes 5*s*, 5*p*, 6*s*, 5*d*, and 4*f* electrons explicitly and is henceforth referred to as the 4*f*-valence potential. An alternative potential for Ce includes one 4*f* electron in the core to explicitly define Ce^3+^ cations in ceria. This potential is labeled as Ce_3 (11 May 2000) in the VASP pseudopotential repository and is henceforth referred to as the 4*f*-core potential in this article. The convergence criterion used for the electronic structure self-consistent field was 5 × 10^–6^ eV. The geometries were relaxed until forces acting on the atoms which were allowed to displace were smaller than 0.05 eV Å^–1^.

The PW91^47^ generalized gradient approximation (GGA) exchange–correlation functional was used. To properly describe electron localization on 4*f* states of Ce atoms when using the 4*f*-valence potential and partially correct the self-interaction error typical of semi-local exchange–correlation functionals, we employed the GGA+*U* approach.^48^ This introduces an energy penalty on states with partial occupation of Ce4*f* orbitals. For consistency with previous studies dealing with ceria-supported clusters and formation of Ce^3+^ cations,^36,49−51^ we used a *U* value of 4 eV.

As stated in the Introduction section, converging calculations with the desired number and position of Ce^3+^ cations is challenging even when using the GGA+*U* scheme. To overcome this, previous studies have carried out structural relaxations using the 4*f*-core potentials to precondition the structure such that a posterior (and presumably more accurate) calculation with 4*f*-valence potentials converges to the desired electronic state more easily. Often, but not always,^52^ energies resulting from this preconditioning procedure have been discarded in favor of those obtained in posterior calculations without constraining a selected number of 4*f* electrons to the corresponding Ce cores. However, the adequacy of the 4*f*-core potentials for describing the relative energies, electronic structure, and chemical properties of ceria-based systems has not been addressed. Therefore, in this work, we evaluate properties of ceria-supported Pt particles in various electronic states differing in the number and position of the Ce^3+^ cations obtained both with and without 4*f*-core potentials and propose a general approach to evaluate EMSI directly via calculations using 4*f*-core potentials.

We calculated the relative energies of different electronic states, the corresponding atomic charges (based on a Bader analysis^53^) on the metal atoms of the supported Pt particle, and the reactivity of these atoms toward adsorption of catalytically relevant species. Using the 4*f*-core potentials straightforwardly allows us to obtain any electronic state and circumvent challenging convergence issues regarding the localization of Ce4*f* electrons. These convergence problems are particularly prevalent for metastable minima, for which Ce4*f* orbitals often change their occupation during structural relaxation.

Another advantage of using Ce4*f*-core potentials is that spin-polarized calculations are not required to describe the localized 4*f* electron fixed in the core. We can therefore characterize the desired electronic states in a computationally efficient way. Spin-restricted calculations, however, disregard such aspects as the magnetization often exhibited by supported metal particles or the magnetic coupling between Ce4*f* electrons. We therefore validated selected results also with spin-polarized calculations.

### 2.2 Structural Models

**Figure 1:**
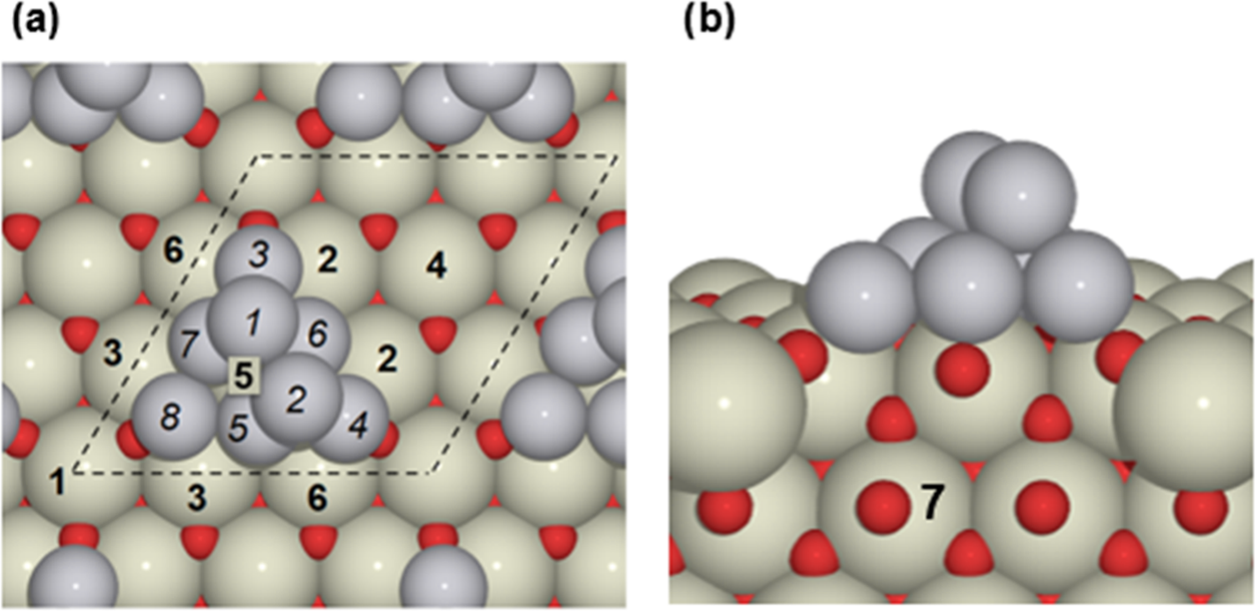
Structural model of the Pt_8_ cluster supported on the CeO_2_(111) surface: (a) top view and (b) side view. Dashed lines delimit the 3 × 3 supercell used. Pt, Ce, and O atoms are depicted in gray, beige, and red, respectively. Numbers in bold label positions of Ce atoms relative to the Pt_8_ cluster. The Ce-5 atom is located below the Pt_8_ cluster. Ce-7 corresponds to a subsurface Ce atom. Symmetrically equivalent Ce atoms have the same label. Numbers in italics label the Pt atoms.

To represent ceria-supported Pt particles, we used a periodic slab model with a Pt_8_ cluster supported on the CeO_2_(111) surface as shown in Figure 1. The CeO_2_(111) slab is formed by 3 O–Ce–O trilayers, atomic positions in the lower of which were kept fixed during structural relaxations. This slab thickness has been shown to be sufficient in previous studies of oxygen vacancy formation on the CeO_2_(111) surface.^10,12^ The experimental lattice parameter of ceria, 5.41 Å,^54,55^ was used to construct the slab. This is 0.08 Å smaller than the equilibrium lattice parameter predicted with the PW91+*U* (*U* = 4 eV) level of theory employed in the present study.

The chosen 3 × 3 supercell size offers a good compromise between the computational cost and the particle density, with distances larger than 5.9 Å between Pt_8_ particles in neighboring cells. This supercell also provides a manageable number of possible configurations with different numbers and positions of Ce4*f* electrons (Ce^3+^), which facilitates their systematic and exhaustive characterization. Namely, for states resulting from one electron transferred to ceria (i.e., with one Ce^3+^ cation formed), there are 6 different electronic states possible corresponding to 6 inequivalent surface Ce^3+^ positions; for two transferred electrons, there are 18 different states; and for three transferred electrons, there are 35 different states. We also carried out benchmark calculations with a larger (and more computationally costly) 4 × 4 supercell (see Figure S1) to evaluate how the results change upon reducing the Pt particle coverage and increasing the distance between the particles in neighboring cells (from ∼6 to ∼10 Å). However, all results presented henceforth correspond to the 3 × 3 slab model of ceria unless explicitly stated otherwise.

## 3 Results and Discussion

### 3.1 Relative Energies of the Electronic States of Pt_8_/CeO_2_(111)

We begin by evaluating the effect of combining Ce4*f*-core and Ce4*f*-valence potentials (the scheme is hereafter called 4*f*-core) instead of using only 4*f*-valence potentials (4*f*-valence scheme) on the calculated relative energies of different electronic states of CeO_2_(111)-supported Pt_8_.

First, the total energy *E*^4*f*-*core*^ of all possible electronic states with a different number (from 0 to 3) and position of the Ce4*f* electrons is calculated using the 4*f*-core potential for the atoms selected to be Ce^3+^ cations. This provides relative energies for states with the same number of Ce^3+^ cations but does not allow comparing states with different number of atoms described with the 4*f*-core potential. Since previous modeling studies addressing reduced ceria surfaces and nanoparticles^56−58^ have shown that the reduction of surface Ce^4+^ cations is preferred to those in more bulk-like positions, we have sampled electronic states with Ce^3+^ cations in the outermost Ce layer only. However, to validate this approach, we also evaluated the stability of an electronic state with a single Ce^3+^ cation in a subsurface position.

**Figure 2:**
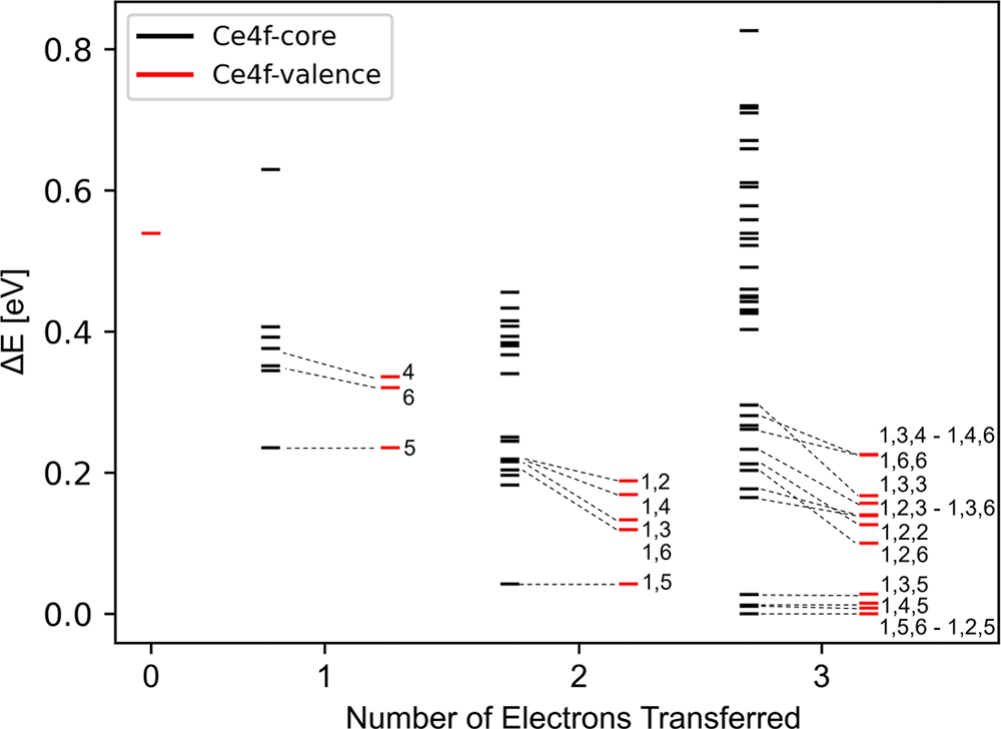
Relative energies Δ*E*^4*f*-valence^ and Δ*E*_rel_^4*f*-core^ of all possible electronic states calculated for the Pt_8_/CeO_2_ system with 0 to 3 electrons transferred from Pt_8_ to the CeO_2_ support. Black bars correspond to Δ*E*^4*f*-*core*^ values obtained using the Ce4*f*-core potential (with Ce4*f* electrons in the fixed core of selected Ce atoms) and spin-restricted calculations. Red bars correspond to Δ*E*^4*f*-valence^ values obtained using the Ce4*f*-valence potential only (without core Ce4*f* electrons) and spin-unrestricted calculations. Numbers next to red bars specify positions of the Ce^3+^ cations as labeled in Figure 1. Dashed lines connect values for states with Ce^3+^ cations in the same positions calculated using 4*f*-core and 4*f*-valence schemes. All calculated relative energy values are provided in Table S1.

To establish the relative energy of states with different number of transferred electrons and to assess the relative energies calculated with the 4*f*-core potential, we calculated the total energy *E*^4*f*-valence^ of several electronic states using the 4*f*-valence potentials only. All calculated relative energies Δ*E* are presented in Figure 2, where black and red lines indicate values calculated with (Δ*E*^4*f*-core^) and without (Δ*E*^4*f*-valence^) the Ce4*f*-core potential, respectively. Δ*E*^4*f*-valence^ values are calculated with respect to the *E*^4*f*-valence^ of the most stable state found (*E*_min_^4*f*-valence^), i.e., the global minimum according to the 4*f*-valence scheme. Δ*E*_*i*_^4*f*-core^ of a state *i* with n transferred electrons is calculated as
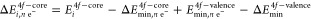
where  is the lowest *E*^4*f*-core^ among states with the same number n of Ce4*f* electrons and  is the Δ*E*^4*f*-valence^ of that same state. We recall that it is not always possible to converge to the desired electronic state without using 4*f*-core potentials, particularly for unstable states. For such cases, calculations often instead converge to other states with a more favorable number or position of the Ce4*f* electrons.

The data in Figure 2 and Table S1 reveal that the energy differences between electronic states strongly depend on both the number and position of the Ce4*f* electrons. Δ*E*^4*f-*core^ for the same number of Ce^3+^ can be as high as 0.82 eV (for 3 transferred electrons), and the highest and the lowest energies calculated among all possible Ce4*f* electron distributions correspond to states with three transferred electrons. The most stable state is that with 3 transferred electrons from the Pt_8_ particle to the ceria surface. The most stable states with two, one, and zero transferred electrons have Δ*E*^4*f*-valence^ of 0.03, 0.22, and 0.54 eV, respectively. The presence of several quasi-degenerate states within ∼0.2–0.3 eV and differing in the number and position of Ce^3+^ cations suggest a facile mobility of electrons between different Ce ions and the supported Pt_8_ particle. EMSI for this system are thus expected to involve a significant dynamic behavior, similarly to ceria-supported Pt atoms^37^ or Au particles.^59^ We note that the *E*^4*f*-core^ of the electronic state with a single Ce^3+^ cation in a subsurface position is 0.39 eV larger than the *E*^4*f*-core^ of the most stable state found with one transferred electron. This implies that the sampling of electronic states with Ce^3+^ cations in subsurface positions is not necessary.

The relative energies Δ*E*^4*f*-valence^ calculated with unrestricted spin and Ce4*f*-valence potentials (red bars in Figure 2) are smaller than the corresponding energies Δ*E*^4*f*-core^ calculated with restricted spin using Ce4*f*-core potentials (black bars in Figure 2). The larger energy differences obtained with the Ce4*f*-core potentials may be attributed to disregarding the magnetic coupling between the Ce4*f* electrons or to the slightly stronger ionic character of Ce^3+^ cations when described with a fixed 4*f* electron. Apparently, the fixed electron cannot participate in the polar covalent Ce–O bonding, leading to a slightly higher atomic charge (by ∼0.01–0.03 |e|) and stronger electrostatic interaction. However, the 4*f*-core and 4*f*-valence approaches agree in the most stable electronic states, with changes (or crossings) in the stability order found for states mostly with large Δ*E*^4*f*-core^ and Δ*E*^4*f*-valence^ values (Figures 2 and S2). We also note that considering spin polarization has little effect on calculated Δ*E*^4*f*-core^ values, with differences ≤0.02 eV per transferred electron (see Table S1). The Δ*E*^4*f*-valence^ values calculated with the 4 × 4 supercell are in good agreement with those obtained with the 3 × 3 supercell (Figure S3, Table S2). Using the 4 × 4 supercell, energy differences between the most stable electronic states with two transferred electrons and the states with one and zero transferred electrons are 0.23 and 0.54 eV, respectively. These energy differences are 0.22 and 0.55 eV, when using the 3 × 3 supercell.

From the calculated relative energies, some trends can be identified regarding the formation of Ce^3+^ cations in Pt_8_/CeO_2_(111). Electron transfer from the supported Pt_8_ cluster is favored to Ce^4+^ cations in closer contact to the cluster (see labels in Figures 1 and 2). We attribute this to the additional space created around Ce atoms upon the formation of Pt–O bonds. The latter weaken and elongate the corresponding Ce–O bonds, thus facilitating the presence of Ce^3+^ cations, which have characteristically larger ionic radii than Ce^4+^ (see Table S3). For the most favorable position for forming Ce^3+^ (position 5, Figure 2), the 3 Ce–O bonds at the surface extend to 2.45 Å *vs* 2.35 Å of Ce–O in the bare surface. Ce-1 is another very favorable position for hosting a 4*f* electron because of its proximity to three neighboring Pt_8_ clusters resulting in extended Ce–O bonds. The most stable Ce^3+^ distributions for states with more than one transferred electron also involve this position. We also note that the Ce^3+^–O distances predicted with the 4*f*-core or 4*f*-valence approaches are in very good agreement (see Table S3). This is unsurprising given that the 4*f*-core potential of Ce has been traditionally used not to directly model desired polaronic states in reduced ceria but rather to precondition the structures in a prerelaxation calculation. This facilitates the convergence to the desired electronic state in a posterior relaxation with the 4*f*-valence approach.

Ce^3+^ cations also avoid being immediate neighbors, in agreement with previous work^10,12^ dealing with the characterization of oxygen vacancies in reduced ceria. For example, once a Ce^3+^ is formed in position Ce-5 (below the cluster), reducing one of the neighboring Ce^4+^ cations (Ce-2 or Ce-3) is less favorable than reducing Ce^4+^ cations in other sites.

The calculated relative energies indicate that Ce4*f*-core potentials can be used to quite reliably identify stable electronic states in systems involving reduced ceria. Considering the more facile convergence to the desired electronic state and the possibility to use spin-restricted calculations, this approach enables the systematic and computationally efficient characterization of EMSI.

### 3.2 Effect of EMSI on the Electronic Structure of Pt_8_ Supported on CeO_2_(111)

To evaluate the EMSI effect on the electronic structure of the CeO_2_(111)-supported Pt_8_ cluster, we compare the projected density of states (pDOS) and atomic Bader charges for several electronic states described in the previous section differing in the number and position of Ce^3+^ centers (i.e., in the number of electrons transferred from the Pt_8_ cluster and the position of the Ce^4+^ cations that the transferred electrons reduce). We also evaluate the differences between results obtained using the 4*f*-core potential (to describe Ce^3+^ centers) and spin-restricted calculations to those calculated with spin polarization with the 4*f*-valence potential. However, we note that the spin-restricted and spin-unrestricted calculations using the 4*f*-core potential yield practically identical pDOS for the supported Pt_8_ clusters (see Figure S4).

**Figure 3:**
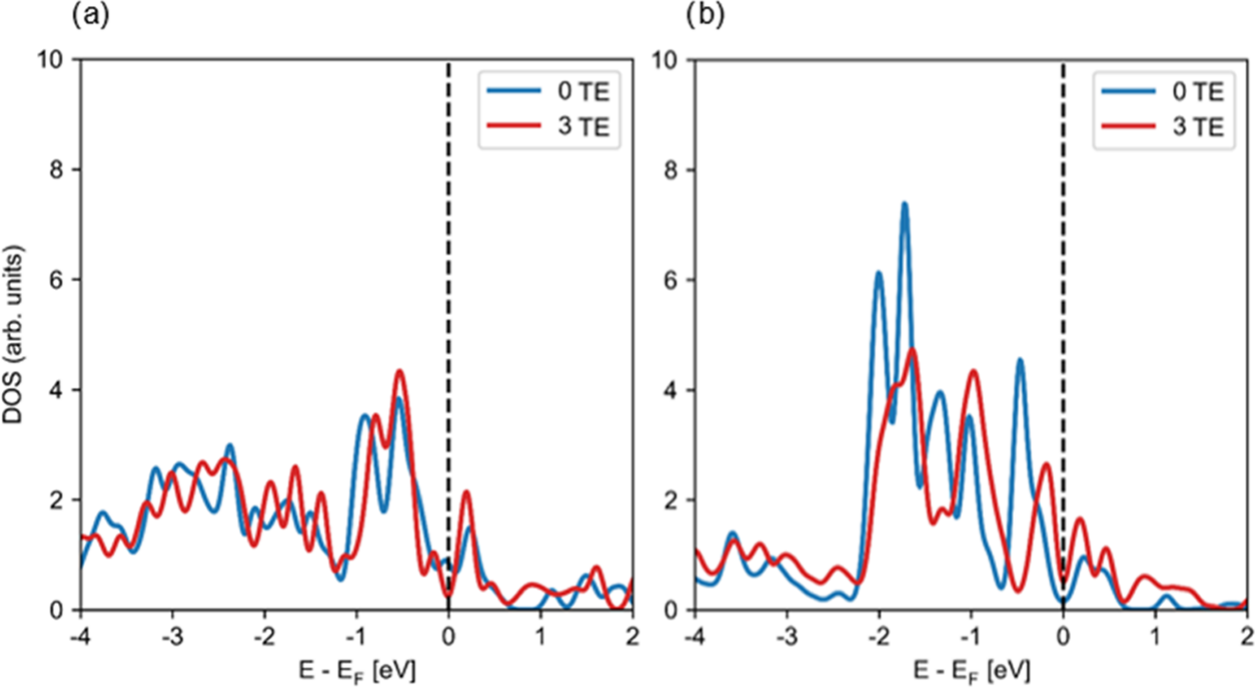
Density of states (DOS) projected on (a) Pt-1 second-layer and (b) Pt-8 interface atoms (as labeled in Figure 1) of the Pt_8_ cluster supported on the CeO_2_(111) surface for the most stable electronic state (3 electrons transferred) and the state without electrons transferred from the metal to the oxide. The data are calculated spin-unrestricted using the 4*f*-valence potential only. Energies are shifted with respect to the Fermi level, which is indicated by a vertical dashed line.

The added pDOS of all atoms of the Pt_8_ cluster for the states with 0, 1, 2, and 3 transferred electrons is shown in Figure S5, although a straightforward analysis of the differences is challenging. We therefore focus instead on the changes in pDOS projected on individual Pt atoms upon the transfer of electrons from Pt_8_ to CeO_2_(111), namely, an interface Pt atom and a second-layer Pt atom (Pt-8 and Pt-1 in Figure 1, respectively). Results of spin-polarized calculations with Ce4*f*-valence potentials in Figure 3 allow us to compare the state with no transferred electrons to the most stable state obtained featuring three transferred electrons and reduced Ce-1, Ce-5, and Ce-6 centers.

The pDOS of the interface atom and the second-layer atom are different regardless of the electronic state, which reflects an inhomogeneous charge distribution within the supported Pt_8_ cluster similarly to ceria-supported Au particles.^59^ Interestingly, there are only little differences in the pDOS of the second-layer Pt (Pt-1, Figure 3a) between the two electronic states, whereas the intensities and positions of the pDOS peaks differ notably for the interface Pt atom (Pt-8, Figure 3b). Therefore, the charge redistribution upon electron transfer is also inhomogeneous and has a stronger effect on interface atoms. As we show below, this is also reflected in the atomic charges and the chemical properties of these Pt atoms.

We have also calculated the pDOS for systems with the same number but different positions of Ce^3+^ cations, revealing that the exact location of the Ce4*f* electrons has a small effect on the electronic structure of the supported particles (see Figure S6). Thus, despite the effect of such positions on the stability of the Pt_8_/CeO_2_(111) models, the electronic structure of supported Pt particles is noticeably affected only by the number of transferred electrons.

Integration of the DOS projected on different orbitals of the atoms in the Pt_8_ particle reveals a lower number of occupied *d* states but a higher number of occupied sp states (see Table S3) upon electron transfer to the oxide surface. This indicates that electrons are transferred from Pt *d* orbitals to ceria, with a concomitant back-donation of electrons to the Pt sp states. The values for the integrated pDOS of all states of Pt evolve erratically with the number of Ce^3+^ centers, with an abnormally large value for the state with two transferred electrons. Despite this, atomic charges on the Pt_8_ cluster evolve as expected and become progressively larger upon the transfer of every additional electron (*vide infra*).

**Figure 4:**
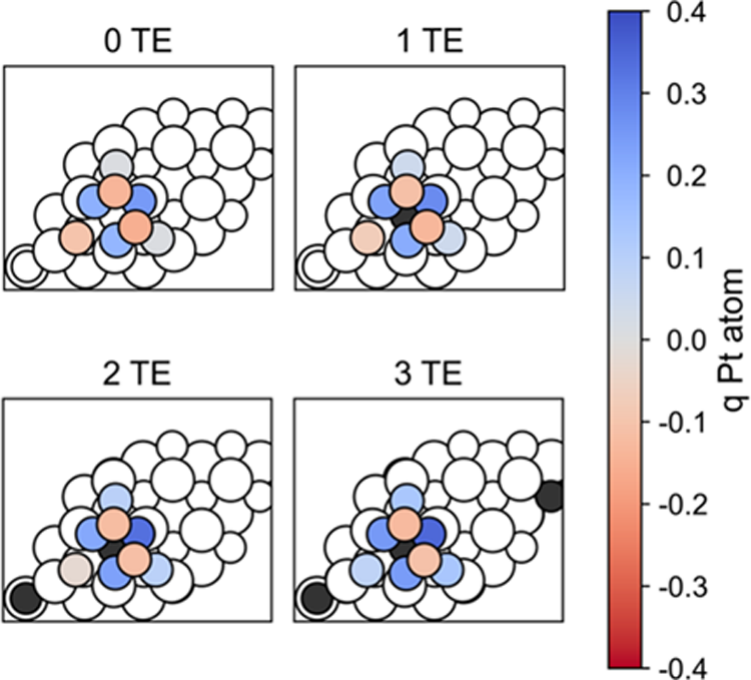
Bader charges (q) of atoms in the Pt_8_ cluster supported on CeO_2_(111) for most stable electronic states with 0–3 transferred electrons (TE) from the Pt cluster to the oxide. Pt atoms are colored according to their Bader charges, as indicated in the colorbar: red—negative charge and blue—positive charge. Black circles depict Ce^3+^ cations. The corresponding values are also given in Table S4.

The inhomogeneous charge distribution and nonuniform effect of electron transfer on interface and second-layer atoms of the Pt cluster is also reflected on the corresponding atomic Bader charges (see Figure 4 and Table S4). For all states, interface Pt atoms are more oxidized (i.e., carry larger positive Bader charges) than second-layer Pt atoms. Comparison of Bader charges of atoms with different coordination should, however, be carried out with care. The larger Bader volume assigned to less coordinated atoms can lead to more negative Bader charges on them, even for systems where a polarization is not expected. It is more reliable to analyze variations of the Bader charges upon a given process. For example (Table S4), the total charge of the Pt_8_ cluster is, as expected, larger for more oxidized states. The electronic states with zero, one, two, and three transferred electrons exhibit total Pt_8_ cluster charges of 0.25, 0.49, 0.70, and 1.03 |e|, respectively. The majority of this 0.78 |e| variation involves interface Pt atoms, whose charge increases by 0.06 to 0.21 |e|. In contrast, the charges of the second-layer atoms Pt-1 and Pt-2 remain nearly constant. Thus, EMSI and related electron transfer in CeO_2_-supported Pt particles are expected to affect the chemical properties mainly of the interface metal atoms. Similar to what was observed for the pDOS, the Bader charge of the Pt atoms is also hardly sensitive to the position of the Ce^3+^ centers and mainly depends on the number of transferred electrons (Table S5). The electronic structure of the supported Pt particle is also insensitive to the particle coverage decrease, with almost identical pDOS (see Figure S7) and Bader charges (see Table S6). Thus, the shorter distances between Pt particles have a negligible effect on the relative stability of different electronic states, the atomic charges of the Pt atoms, and the orbital energies of the Pt particle.

The effect of electron transfer is also reflected in structural changes of the Pt_8_/CeO_2_(111) model. More positive charges of the Pt atoms at the interface lead to a shortening of the corresponding Pt–O bonds (Table 1). This is attributed to strengthening of such bonds induced by the decrease in occupation of antibonding Pt5*d* states upon partial oxidation. As we show below, these electronic effects also lead to stronger bonding with different adsorbates.

**Table 1:** Pt-n–O Bond Lengths between the Pt-n Atom and the Nearest O Atoms of the CeO_2_(111) Surface Calculated for Electronic States with 0 to 3 Transferred Electrons (TE) from Pt_8_ to CeO_2_^a^

Pt-n–O [Å]	0 TE	1 TE	2 TE	3 TE
Pt-3	2.07	2.06	2.02	2.00
Pt-4	2.07	2.06	2.02	2.00
Pt-5	2.02	2.02	2.02	2.00
Pt-6	2.01	2.00	1.99	1.98
Pt-7	2.02	2.02	2.02	2.00
Pt-8	2.09	2.08	2.04	2.02

a Numbers *n* are the labels of Pt atoms in Figure 1.

The strengthening of the Pt–O bonds upon increasing cationic character of Pt atoms is consistent with the relative energies reported in Figure 2, where the states with more oxidized Pt (less occupied antibonding Pt5*d* orbitals) are more stable than the states with more metallic Pt.

Finally, using the Ce4*f*-core potential to describe Ce^3+^ cations and spin-restricted calculations has a negligible effect also on the Bader charges of the Pt_8_ cluster. This, together with the above results on pDOS of Pt atoms, justifies that the more computationally efficient 4*f*-core approach can reliably describe the electronic structure of different states of ceria-supported Pt particles.

### 3.3 Chemical Properties of the Pt_8_ Cluster in Different Electronic States

We have characterized the effects of EMSI on the reactivity of different sites of the ceria-supported Pt_8_ particle. In particular, we considered the adsorption of common adsorbates CO, H_2_O, OH, and H calculated at two DFT levels, i.e., with and without describing Ce^3+^ cations with the 4*f*-core potential. The adsorption energies E_ads_ are defined as
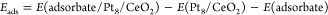
where *E*(adsorbate/Pt_8_/CeO_2_) and *E*(Pt_8_/CeO_2_) are the total energies of the models with and without the adsorbate, respectively. *E*(adsorbate) is the energy of the gas-phase adsorbate species, i.e., *E*(CO), *E*(H_2_O), *E*(OH), or 1/2E(H_2_). *E*_ads_ values are calculated for adsorbate/Pt_8_/CeO_2_ and Pt_8_/CeO_2_ states with the same number and positions of Ce^3+^ cations, i.e., assuming that the oxidation state of the Pt_8_ particle and underlying oxide is preserved upon adsorption. This allows examining the chemical properties for every individual electronic state under scrutiny. We considered the adsorption on the Pt-8 interface and Pt-1 second-layer atoms, for the electronic states with zero to three transferred electrons probing on-top adsorption geometries. For CO, OH, H_2_O, and H, the on-top site of a second-layer atom is the most favorable adsorption site for the electronic state without any transferred electrons, with bridge or hollow sites being less stable. For interface Pt atoms, the on-top site is the most favorable adsorption site for CO, OH, and H_2_O, whereas for H, the bridge site between an interface and a second-layer atom is more favorable. Nevertheless, we only consider on-top sites in our comparison to suppress effects emerging from differences in site symmetry. Note that we locally reoptimize (relax) the structure for every combination of electronic state, site, and adsorbate.

**Figure 5:**
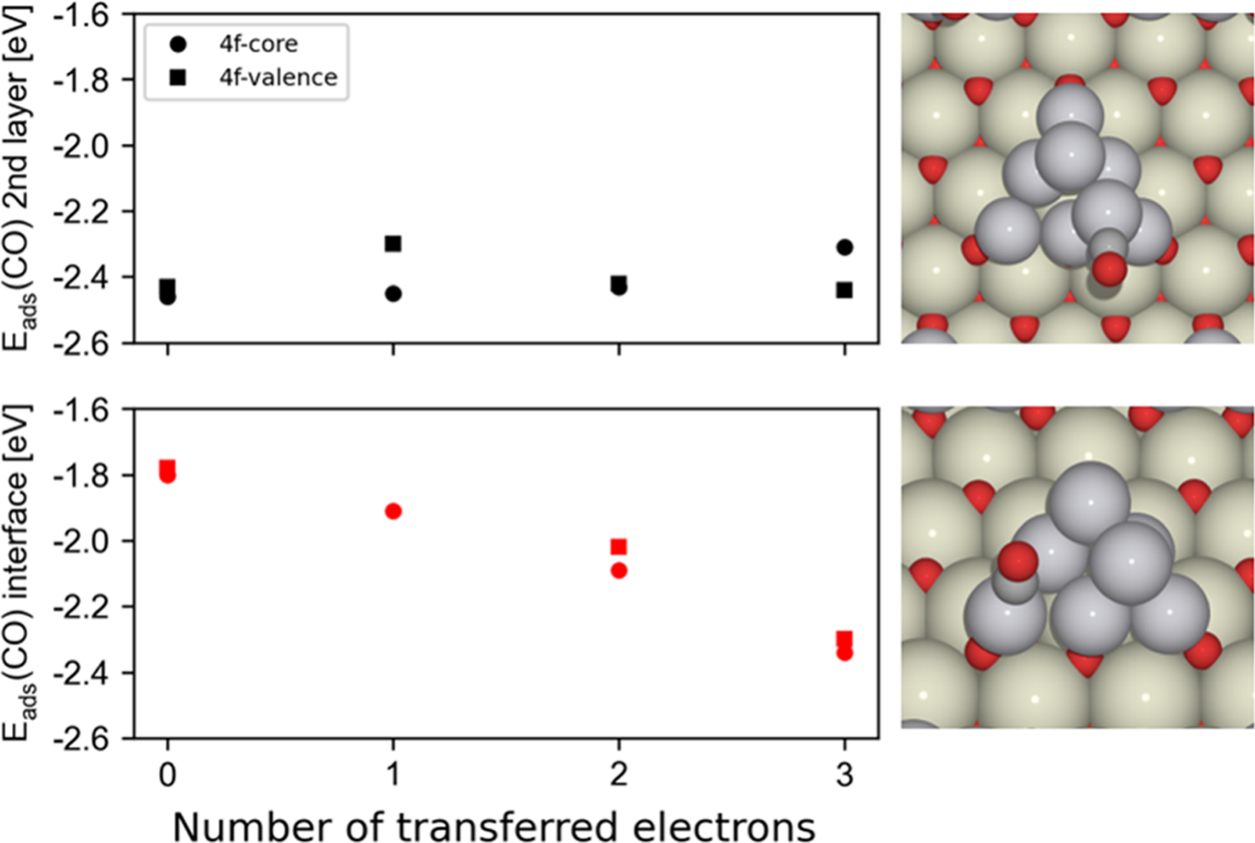
CO adsorption energies on the interface (Pt-8) and second-layer (Pt-1) atoms of the Pt_8_ cluster supported on CeO_2_(111) for the electronic states with 0–3 electrons transferred from Pt_8_ to CeO_2_. Data calculated using the 4*f*-core, spin-restricted approach are shown by circles, and those calculated using the 4*f*-valence, spin-unrestricted method are shown by squares. The two evaluated CO adsorption sites (on-top interface and on-top second layer) are illustrated in the right-hand panels, which show the relaxed structures for the electronic state without any transferred electrons.

The adsorption energies of a single CO molecule on-top of the two selected Pt-8 and Pt-1 sites are shown in Figure 5 and Table S7, revealing clear differences for different sites, as well as a markedly different evolution with the number of transferred electrons. For the electronic state without any transferred electrons, CO adsorption is 0.65 eV stronger on the second-layer site, whereas the adsorption energies are almost identical for the electronic state with three transferred electrons. In agreement with the more pronounced changes in the electronic structure of the interface Pt atoms due to Pt_8_–CeO_2_ charge transfer, the reactivity of the interface sites is also more strongly modified upon oxidation of the particle and concomitant reduction of the ceria surface. Thus, not unexpectedly, EMSI mainly affect the chemical properties of the interface metal atoms, which could be tuned by controlling the number of transferred electrons.

CO adsorption energies obtained using the 4*f*-core, spin-restricted approach agree well with the energies using the 4*f*-valence and spin-unrestricted level of theory. *E*_ads_ trends are well reproduced, and the *E*_ads_ differences between these two approaches range only from 0.02 to 0.10 eV. These *E*_ads_ differences are mainly due to treating the Ce4*f* electron as a part of the core, whereas the energy differences between the spin-restricted and spin-unrestricted calculations are minor (see Table S7). Some states, such as that for one transferred electron and CO adsorbed on the interface site, can only be obtained using the 4*f*-core potential, with calculations converging to other electronic states when using the 4*f*-valence potential only. In turn, states without any transferred electrons can obviously only be obtained without the 4*f*-core potential, and we therefore just use spin-restricted calculations with the 4*f*-valence potentials to complete the 4*f*-core series. Finally, the just discussed *E*_ads_(CO) values calculated with the ceria supercell 3 × 3 change only moderately, by up to 0.1 eV, when using a larger supercell 4 × 4 (see Table S8).

**Figure 6:**
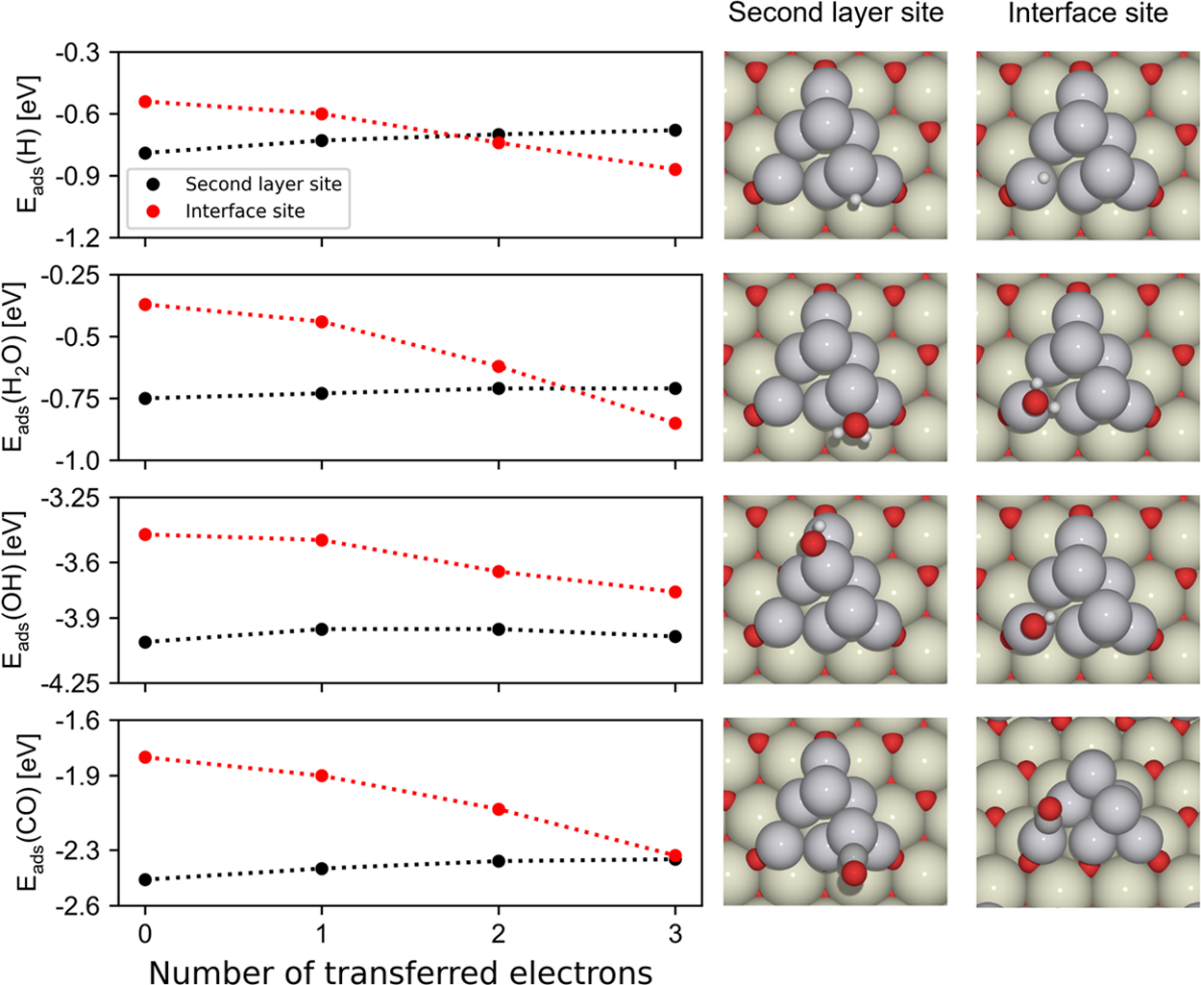
Adsorption energies of H, H_2_O, OH, and CO adsorbates on Pt_8_/CeO_2_(111) for the most stable electronic states with different number of electrons transferred from the metal cluster to the oxide support. The black and red markers correspond to adsorption energies of adsorbates in on-top positions at the second-layer and interface sites of Pt_8_, respectively. The two evaluated sites for every adsorbate (on-top interface and on-top second layer) are illustrated in the right-hand panels, which show the relaxed structures for the electronic state without any transferred electrons. The corresponding energy values are also reported in Table S9.

The good established performance and accuracy of the spin-restricted calculations using the 4*f*-core potential for describing Ce^3+^ cations open a way to reliably and systematically evaluate E_ads_ trends across different adsorbates (H, OH, H_2_O), sites (interface and second layer), and electronic states (from zero to three transferred electrons); see Figure 6 and the data in Table S9.

In the absence of transferred electrons, adsorption on the interface site is weaker than on the second-layer site for all adsorbates considered in the present work. As was the case for the adsorbed CO, adsorption of H, H_2_O, and OH on the interface site becomes stronger upon electron transfer from the metal particle to the ceria surface, whereas adsorption energies on the second-layer site remain almost unchanged. The different evolution of the adsorption strength on the interface and second-layer sites with electron transfer even leads to an inversion in preferred adsorption site for H_2_O and H. Thus, EMSI significantly affect adsorption properties of interface sites for various adsorbates. More specifically, the variation from zero to three transferred electrons strengthens the adsorption of H, H_2_O, OH, and CO by 0.3, 0.5, 0.3, and 0.5 eV, respectively. This stronger adsorption can also be considered as a pronounced stabilization upon exposure to reactants of electronic states with oxidized Pt *vs* less oxidized states of Pt. Ceria-supported Pt particles are therefore expected to donate more electrons to the oxide support when adsorbates interact with interface atoms of the Pt particles. Surprisingly, the bond distances between the adsorbate and Pt and between atoms of the adsorbate (e.g., the C–O bond) are not affected by the number of transferred electrons. This suggests that the effect of electron transfer on the adsorption energies is to a significant extent due to a peculiar stabilization of some electron configurations in the presence of an adsorbate. As discussed below, we hypothesize that electrostatic interactions between Ce^3+^ centers and the interface Pt atom play an important role.

The demonstrated dependence of the adsorption properties of Pt atoms at the Pt/CeO_2_ interface on the number of transferred electrons indicates that the affinity of these sites toward reactants and intermediates can be tuned by controlling the reducibility of the ceria support. More reducible ceria-based supports such as ceria nanoparticles^9,11^ or some doped ceria materials^60−62^ will lead to more oxidized Pt particles and, therefore, to stronger interaction with the interface Pt atoms. In turn, ceria supports with a high concentration of oxygen vacancies (i.e., very reduced) and saturated with Ce4*f* electrons would less favorably accept electrons from Pt, stabilizing more reduced Pt states and leading to weaker interactions between adsorbates and interface Pt atoms. A similar picture was reported for ceria-supported Au particles, where reduction of ceria led to charge redistribution and the transfer of electrons back to the supported Au particles.^59^

We have shown above that the electronic structure (pDOS and Bader charges) of both the interface and second-layer Pt sites essentially does not depend on the position of Ce^3+^ centers (only on the concentration of the latter). Interestingly, CO adsorption energies at the interface Pt site noticeably depend on the individual Ce^3+^ positions (see Figure S8a and b). We attribute this to the fact that Pt atoms donate a significant amount of charge to form Pt–CO bonds, leading to more positively charged Pt atoms. Since the electrostatic repulsion of such Pt^δ+^ atoms is weaker from Ce^3+^ than from Ce^4+^ cations, it stabilizes the CO–Pt^δ+^–Ce^3+^ motif *vs* the CO–Pt^δ+^–Ce^4+^ one. This effect is stronger for the 4 × 4 supercell, where adsorption energy differences for states with different Ce^3+^ positions can be as high as 0.34 eV (Figure S8c and d).

Inhomogeneous charge distributions have been reported for other transition-metal particles (Ni, Cu, Au) supported on reducible oxides such as CeO_2_ and TiO_2_,^29,30,63,64^ where interface atoms experience more pronounced charge depletion than those farther away from the support. CO adsorption on ceria-supported Cu particles was also calculated to be site-sensitive, with an adsorption energy of 0.72 eV for Cu^0^ and 0.79 eV for Cu^+^ sites.^63^ A similar trend favoring adsorption on cationic metals was found for Au atoms on CeO_2_, where the adsorption energy of CO is 2.48 eV on Au^+^ sites and 3.24 eV on Au^3+^ sites.^34^ For Pt_8_ particles supported on CeO_2_ nanoparticles, CO adsorption was reported to be weaker on interface sites in contact with ceria,^65^ in line with the present work. This supports previous observations that vibrational frequencies of CO on small Pt clusters supported on nonreducible oxides are dependent on the oxidation state of the adsorption site.^66^ The strengthening of CO–Pt interactions on the interface site of the ceria-supported Pt_8_ cluster with increased electron transfer to ceria calculated using the 4*f*-valence approach^15^ is in quantitative agreement with the 4*f*-core data in Table S9. Thus, the explanation therein of the quite unexpected trend is also partially applicable to the present work. In particular, they report that the main contribution to the stronger CO adsorption comes from the increased σ-donation to Pt with an unchanged 2π back-donation.^15^ We note in passing that the employed 4*f*-valence approach hindered convergence for H_2_O adsorption on the interface site of ceria-supported Pt_8_ at 2 TE,^15^ which did not present a problem within the present 4*f*-core approach. An opposite dependence of the CO adsorption energy on the charge was calculated for ceria-supported Ni, 1.90 eV on metallic and cluster Ni^0^ and 1.39 eV on single-atom Ni^2+^ sites.^67^

The interplay between adsorption energy and charge distribution in the supported catalyst points to the possibility of tuning the chemical reactivity of oxide-supported metal clusters by controlling the oxidation state of reducible oxide supports. Reducing or oxidizing oxide supports would modify their propensity to accept or donate electrons, thus favoring different adsorption sites in supported clusters. Still, it is not clear yet to what extent this electron transfer process depends on the morphology of the metallic nanostructure (single atoms, clusters, nanoparticles, or monolayers) and the reductive capacity of different facets of reducible supports.^68^

## 4 Conclusions

In this work, we have systematically evaluated the effects of EMSI on the properties of ceria-supported Pt particles. We have identified a plethora of electronic states close in energy but differing in the number of transferred electrons from the Pt particle to the underlying oxide surface and in the position of the reduced Ce^3+^ cations. The most stable electronic state found exhibits three transferred electrons, which is over 0.5 eV more stable than the state without transferred electrons. The stability of 4*f* electrons on different Ce positions is mainly related to the available space around each Ce atom, which is in general increased by the formation of Pt–O bonds.

Despite the effect of the position of the Ce4*f* electrons on the stability of the system, the electronic structure of the Pt atoms of the particle is only noticeably affected by the number of transferred electrons, leading to less filled *d* states and more positive Bader charges. Interaction with the ceria support oxidizes interface atoms more significantly, and the charge redistribution upon transfer of electrons affects mainly these Pt atoms located directly at the metal–oxide interface.

The electronic structure changes of the interface Pt atoms significantly affect their chemical properties, strengthening bonds with all evaluated adsorbates (CO, H_2_O, H, and OH) upon transfer of electrons from Pt to ceria. This strengthening is attributed to less occupied antibonding Pt5*d* orbitals on these sites and partially also to weaker electrostatic interactions between positively charged Pt and nearby Ce^3+^ centers. The different reactivity evolution of the interface and the second-layer sites upon transfer of electrons to the support suggests that different sites can be selectively activated by controlling the state of the ceria support, which would accept fewer electrons from Pt particles when already saturated with Ce4*f* electrons.

Finally, we have demonstrated that the approach used in this work relying on the Ce PAW potential with a fixed core 4*f* electron (i.e., the 4*f*-core potential) to describe selected Ce atoms as Ce^3+^ cations allows one to systematically and reliably probe the electronic structure and reactivity of multiple electronic states emerging from the EMSI in ceria-supported Pt particles. Overcoming the known convergence issues and localization challenges of the Ce4*f* electron with this 4*f*-core approach therefore makes the characterization of such complex interactions in ceria-supported metal species straightforward and computationally efficient.

## Data Availability

The outputs from the calculations underlying this study are openly available in ioChem-BD repository at http://dx.doi.org/10.19061/iochem-bd-6-271.^69^

## Supporting Information Available

Structural model of the Pt_8_/CeO_2_(111) model using a 4 × 4 CeO_2_(111) supercell; tabulated and plotted relative energies of electronic states with different numbers and positions of transferred electrons from Pt_8_ to CeO_2_(111); tabulated Ce–O bond distances of relevant structures; DOS plots for different electronic states of the CeO_2_(111)-supported Pt_8_ cluster; Bader charges and integrated pDOS of the Pt_8_ cluster; tabulated adsorption energy values for CO, H_2_O, H, and OH on Pt_8_/CeO_2_; and illustration of the effect of the positions of reduced Ce^3+^ centers on the stability of adsorbed CO (PDF)
