# Supplementary material for: Systematic Characterization of Electronic Metal–Support Interactions in Ceria-Supported Pt Particles
Source: J Phys Chem C Nanomater Interfaces. 2023 Aug 30;127(36):17700–10. doi: 10.1021/acs.jpcc.3c03383 (PMC10510437; doi:10.1021/acs.jpcc.3c03383)
Supplement: Supplementary file 1 — jp3c03383_si_001.pdf [file jp3c03383_si_001.pdf]

Supplementary information for:

Systematic Characterization of Electronic Metal Support Interactions in Ceria-supported Pt Particles

Pablo Castro-Latorre,[a] Konstantin M. Neyman,[a,b] Albert Bruix[a,*]

[a] Departament de Ciència de Materials i Química Física & Institut de Quimica Teòrica i Computacional (IQTCUB), Universitat de Barcelona, 08028 Barcelona, Spain

[b] ICREA (Institució Catalana de Recerca i Estudis Avançats), 08010 Barcelona, Spain

* Corresponding author: abruix@ub.edu

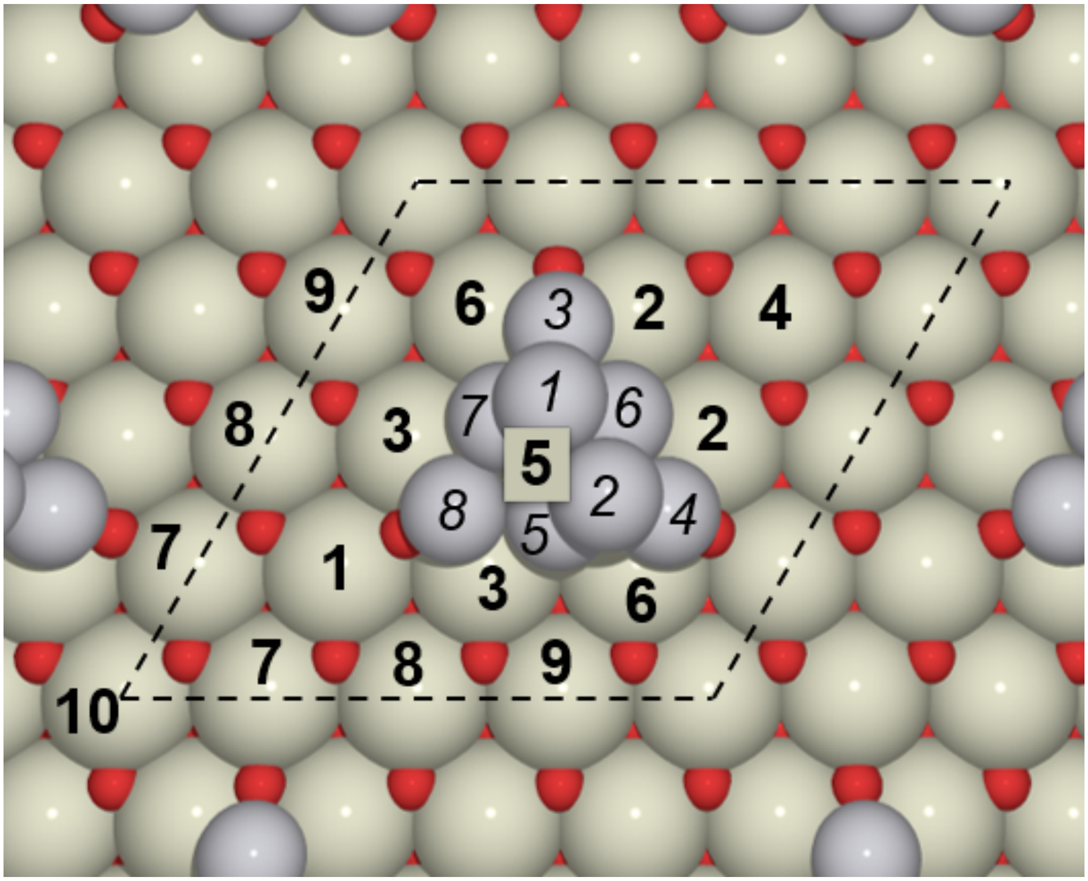

Figure S1: Structural model of a Pt_8 cluster supported on the $CeO_2(111)$ surface. Dashed lines delimit the 4×4 supercell used. Pt, Ce and O atoms are depicted in grey, beige, and red, respectively. Numbers in bold are labels of Ce atoms according to their position relative to the Pt_8 cluster. Ce-5 atom is located below the Pt_8 cluster. Symmetrically equivalent Ce atoms have the same label. Numbers in italics are labels of the Pt atoms.

System	$\Delta E_{rel}^{4f-core}$	$\Delta E_{rel}^{4f-valence}$	System	$\Delta E_{rel}^{4f-core}$	$\Delta E_{rel}^{4f-valence}$
0 TE	-	0.54	3 TE - 1,3,3 (↑↑↑)	0.19	0.15
1 TE - 1	0.32	-	3 TE - 1,3,4 (↓↑↑)	0.25	0.22
1 TE - 2	0.38	-	3 TE - 1,3,5 (↑↑↑)	0.03	0.02
1 TE - 3	0.39	-	3 TE - 1,3,6 (↓↑↓)	0.16	0.13
1 TE - 4 (↑)	0.36	0.32	3 TE - 1,4,5 (↑↓↓)	0.01	0.01
1 TE - 5 (↑)	0.22	0.22	3 TE - 1,4,6 (↑↑↑)	0.21	0.22
1 TE - 6 (↑)	0.34	0.3	3 TE - 1,5,6 (↑↑↑)	0.00	0
1 TE - 7	0.61		3 TE - 1,6,6 (↑↑↑)	0.26	0.16
2 TE - 1,2 (↑↑)	0.20	0.18	3 TE - 2,2,3	0.58	-
2 TE - 1,3 (↑↑)	0.23	0.12	3 TE - 2,2,4	0.68	-
2 TE - 1,4 (↑↑)	0.22	0.16	3 TE - 2,2,5	0.42	-
2 TE - 1,5 (↑↑)	0.03	0.03	3 TE - 2,2,6	0.49	-
2 TE - 1,6 (↓↑)	0.19	0.11	3 TE - 2,3,3	0.57	-
2 TE - 2,2	0.37	-	3 TE - 2,3,4	0.63	-
2 TE - 2,3	0.35	-	3 TE - 2,3,5	0.38	-
2 TE - 2,4	0.40	-	3 TE - 2,3,6	0.50	-
2 TE - 2,5	0.19	-	3 TE - 2,4,5	0.43	-
2 TE - 2,6	0.33	-	3 TE - 2,4,6	0.62	-
2 TE - 3,3	0.42	-	3 TE - 2,5,6	0.42	-
2 TE - 3,5	0.23	-	3 TE - 2,6,6	0.50	-
2 TE - 3,6	0.38	-	3 TE - 3,3,4	0.68	-
2 TE - 4,5	0.17	-	3 TE - 3,3,5	0.46	-
2 TE - 4,6	0.39	-	3 TE - 3,3,6	0.52	-
2 TE - 5,6	0.18	-	3 TE - 3,4,5	0.43	-
2 TE - 6,6	0.37	-	3 TE - 3,4,6	0.67	-
3 TE - 1,2,2 (↑↑↑)	0.17	0.12	3 TE - 3,5,6	0.40	-
3 TE - 1,2,3 (↑↑↑)	0.13	0.13	3 TE - 3,6,6	0.54	-
3 TE - 1,2,4	0.23	-	3 TE - 4,5,6	0.40	-
3 TE - 1,2,5 (↑↑↑)	0.01	0	3 TE - 4,6,6	0.79	-
3 TE - 1,2,6 (↑↑↑)	0.16	0.09	3 TE - 5,6,6	0.39	-

Table S1: Comparison of energy differences ΔE of electronic states calculated using the 4*f*-core scheme and the 4*f*-valence scheme. Energy differences are calculated separately for each number of electrons transferred. The numbers after the number of electrons transferred N TE specify the positions of N surface Ce^{3+} cations according to the labels in Fig. 1. In parenthesis are indicated the spin orientation of every Ce^{3+} cation obtained at the 4*f*valence-su level, where '↑' denotes spin up and '↓' is spin down.

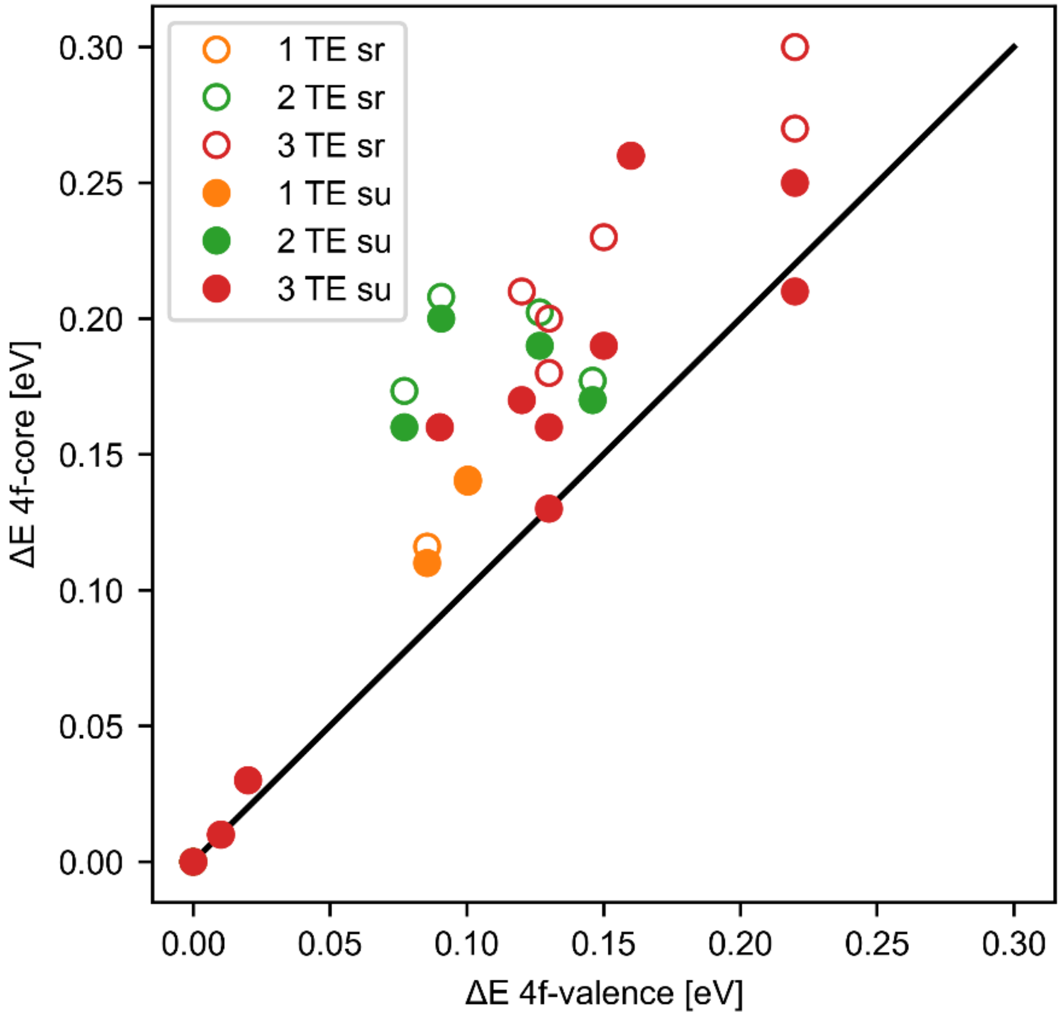

Figure S2: Dispersion of energy difference values obtained with 4*f*-core and 4*f*-valence potentials. Empty circles are energy differences obtained for spin-restricted calculations while solid circles are energy differences obtained for spin-unrestricted calculations. Energy differences are calculated separately for each number of transferred electrons TE.

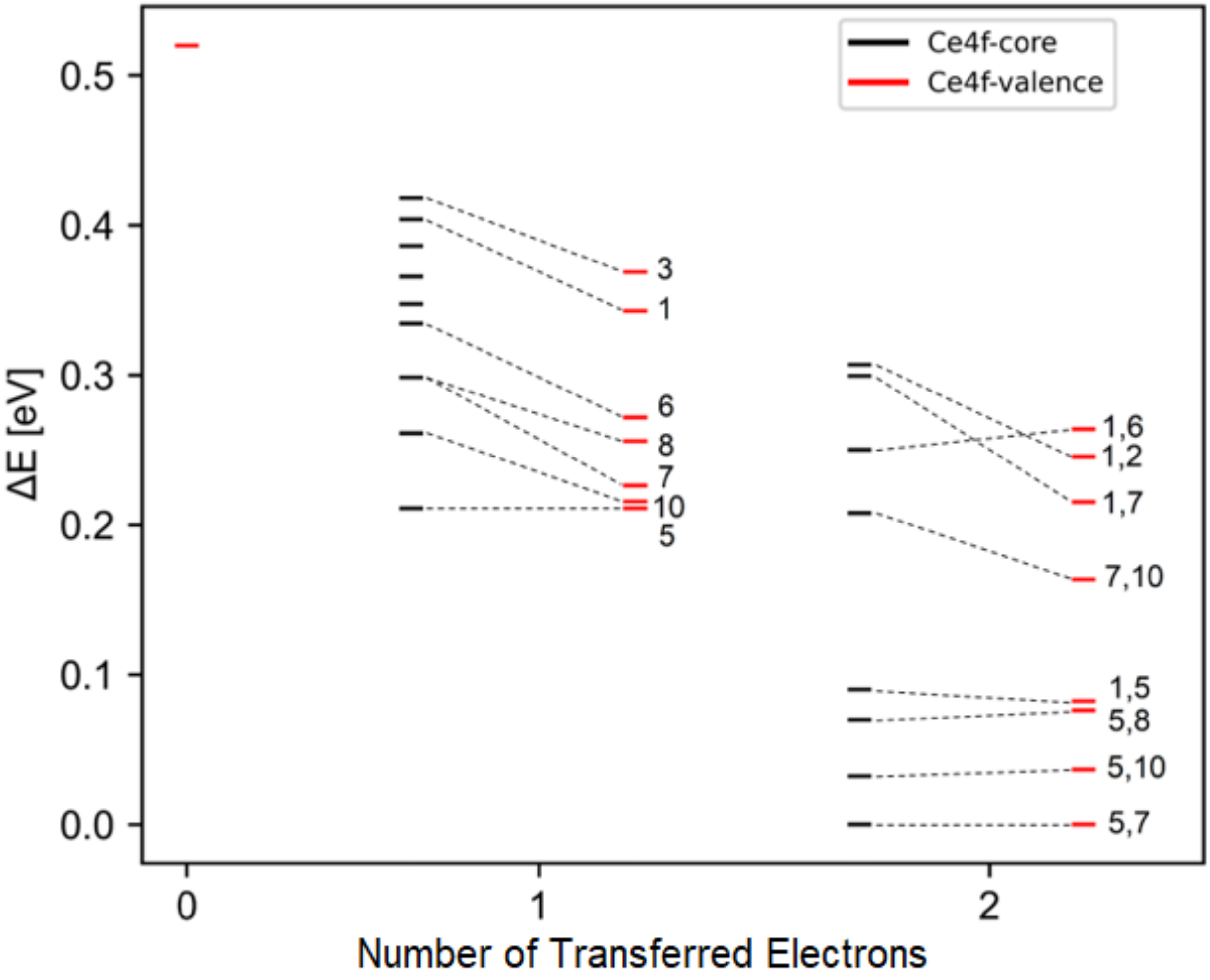

Figure S3: Relative energies $\Delta E^{4f-valence}$ and $\Delta E^{4f-core}$ of all possible electronic states calculated for the Pt_8/CeO_2 system with 0 to 2 electrons transferred from Pt_8 to the CeO_2 support with the supercell size 4×4. Black bars correspond to $\Delta E^{4f-core}$ values obtained using the Ce4*f*-core potential (with Ce4*f* electrons in the core of selected Ce atoms) and spin-restricted calculations. Red bars correspond to $\Delta E^{4f-valence}$ values obtained using the Ce4*f*-valence potential only (without fixed core Ce4*f* electrons) and spin-unrestricted calculations. Numbers next to red bars specify positions of the Ce^{3+} cations as labeled in Figure S1. Dashed lines connect values for states with Ce^{3+} cations in the same positions calculated using the 4*f*-core and 4*f*-valence schemes. All values are also provided in Table S2.

System	$\Delta E^{4f-core}$	$\Delta E^{4f-valence}$
1 TE - 1 (↑)	0.4	0.34
1 TE - 2	0.37	-
1 TE - 3 (↑)	0.42	0.37
1 TE - 4	0.39	-
1 TE - 5 (↑)	0.21	0.21
1 TE - 6 (↑)	0.33	0.27
1 TE - 7 (↑)	0.3	0.23
1 TE - 8 (↓)	0.3	0.26
1 TE - 9	0.35	-
1 TE - 10 (↑)	0.26	0.21
2 TE - 5,10 (↑↑)	0.03	0.04
2 TE - 5,7 (↓↓)	0	0
2 TE - 1,7 (↑↑)	0.3	0.22
2 TE - 5,8 (↓↑)	0.07	0.08
2 TE - 1,5 (↑↑)	0.09	0.08
2 TE - 1,2 (↓↓)	0.31	0.25
2 TE - 1,6 (↑↓)	0.25	0.26
2 TE - 7,10 (↓↑)	0.21	0.16

Table S2: Energy differences of selected electronic states of the 4×4 slab model obtained using the 4*f*-core scheme and the 4*f*-valence scheme. Energy differences are calculated separately for each number of transferred electrons. The numbers after the number of transferred electrons N TE specify the positions of N surface Ce^{3+} cations according to the labels in Fig. S1. In parenthesis are indicated the spin orientation of every Ce^{3+} cation obtained at the 4*f*-valence spin unrestricted level, where '↑' denotes spin up and '↓' is spin down.

Ce^{3+} - O bond distance (surface Ce-O bonds)				Ce^{3+} - O bond distance (sub-surface Ce-O bonds)			
		4f-core	4f-valence			4f-core	4f-valence
0 TE	Ce(5)-O1		2.457	0 TE	Ce(5)-O4		2.363
	Ce(5)-O2		2.455		Ce(5)-O5		2.353
	Ce(5)-O3		2.453		Ce(5)-O6		2.363
	Ce(1)-O1		2.368		Ce(5)-O7		2.369
	Ce(1)-O2		2.374		Ce(1)-O4		2.319
	Ce(1)-O3		2.295		Ce(1)-O5		2.322
	Ce(6)-O1		2.316		Ce(1)-O6		2.317
	Ce(6)-O2		2.299		Ce(1)-O7		2.501
	Ce(6)-O3		2.454		Ce(6)-O4		2.355
					Ce(6)-O5		2.344
1 TE	Ce(5)-O1	2.635	2.630		Ce(6)-O6		2.328
	Ce(5)-O2	2.633	2.623		Ce(6)-O7		2.411
	Ce(5)-O3	2.603	2.592				
				1 TE	Ce(5)-O4	2.445	2.419
2 TE	Ce(5)-O1	2.617	2.617		Ce(5)-O5	2.444	2.424
	Ce(5)-O2	2.627	2.621		Ce(5)-O6	2.445	2.427
	Ce(5)-O3	2.621	2.607		Ce(5)-O7	2.441	2.426
	Ce(1)-O1	2.501	2.494				
	Ce(1)-O2	2.491	2.489	2 TE	Ce(5)-O4	2.444	2.417
	Ce(1)-O3	2.412	2.392		Ce(5)-O5	2.435	2.413
					Ce(5)-O6	2.445	2.425
3 TE	Ce(5)-O1	2.616	2.608		Ce(5)-O7	2.441	2.425
	Ce(5)-O2	2.594	2.573		Ce(1)-O4	2.401	2.381
	Ce(5)-O3	2.658	2.633		Ce(1)-O5	2.409	2.391
	Ce(1)-O1	2.525	2.510		Ce(1)-O6	2.401	2.387
	Ce(1)-O2	2.530	2.517		Ce(1)-O7	2.599	2.596
	Ce(1)-O3	2.512	2.478				
	Ce(6)-O1	2.426	2.394	3 TE	Ce(5)-O4	2.437	2.404
	Ce(6)-O2	2.460	2.419		Ce(5)-O5	2.444	2.414
	Ce(6)-O3	2.638	2.609		Ce(5)-O6	2.404	2.384
					Ce(5)-O7	2.434	2.414
					Ce(1)-O4	2.390	2.384
					Ce(1)-O5	2.363	2.357
					Ce(1)-O6	2.389	2.383
					Ce(1)-O7	2.644	2.654
					Ce(6)-O4	2.448	2.418
					Ce(6)-O5	2.426	2.405
					Ce(6)-O6	2.401	2.375
					Ce(6)-O7	2.485	2.471

Table S3: Ce-O bond distances, comparing 4f-core and 4f-valence potentials for different states with different numbers of Ce^{3+} cations. The left side of the table shows Ce-O distances with surface oxygen atoms and the right-side shows Ce-O distances with sub-surface oxygen atoms bonded to the Ce atom identified by the labels described in Figure 1a. All distances are in Angstrom (Å).

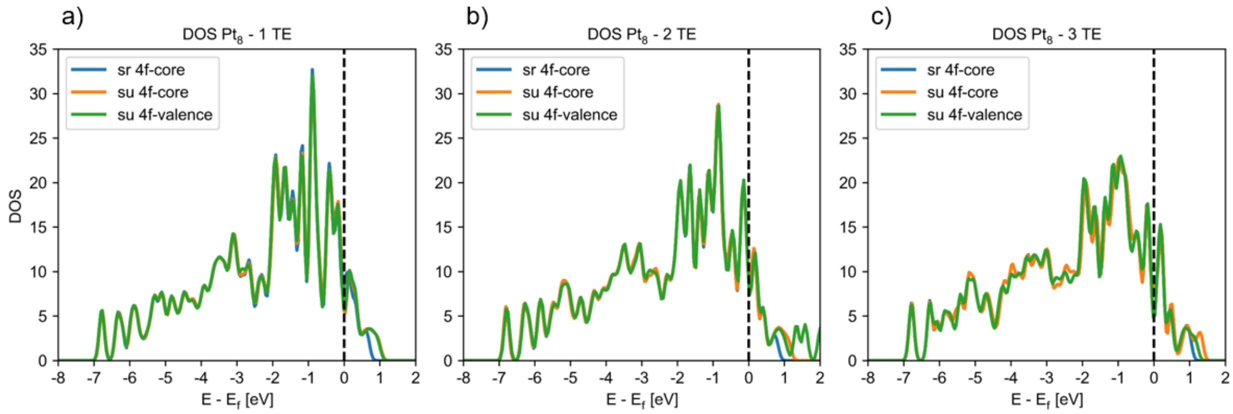

Figure S4: Density of states projected on the Pt atoms of the Pt_8 cluster supported on $CeO_2(111)$ for the most stable electronic states with a) – one, b) – two and c) - three electrons transferred from the cluster. The methodology to calculate the electronic structure is specified by the color of the lines.

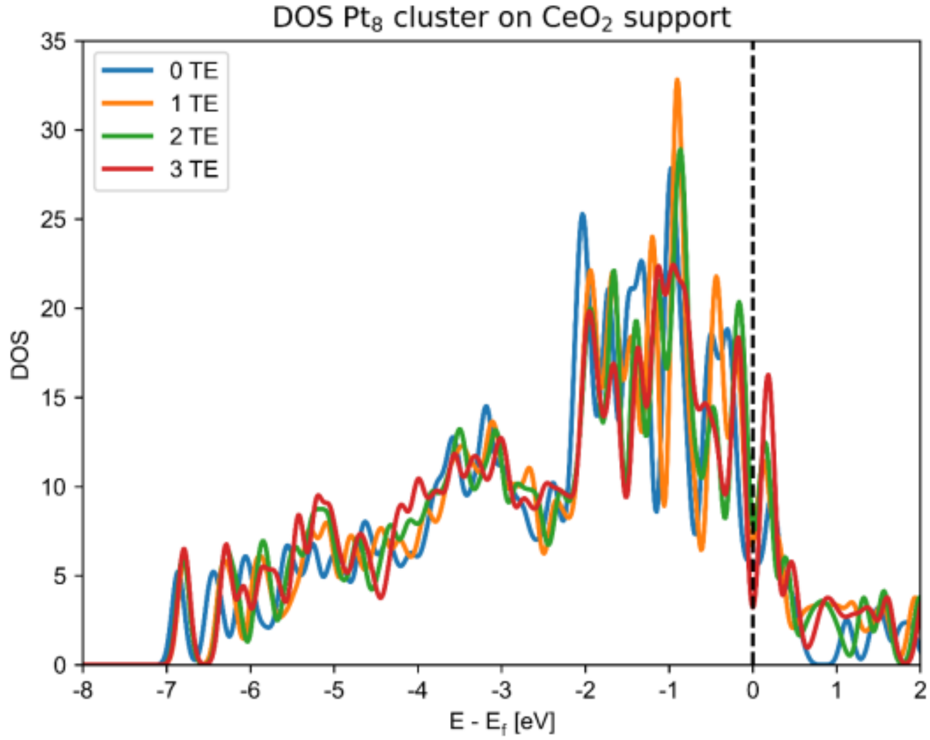

Figure S5: Density of states projected on the Pt atoms of the Pt_8 cluster for 0 to 3 electrons transferred from it, taking the most stable state in each case calculated with the 4f-valence scheme. Energy values are calculated with respect to the Fermi level, marked by a vertical dashed line.

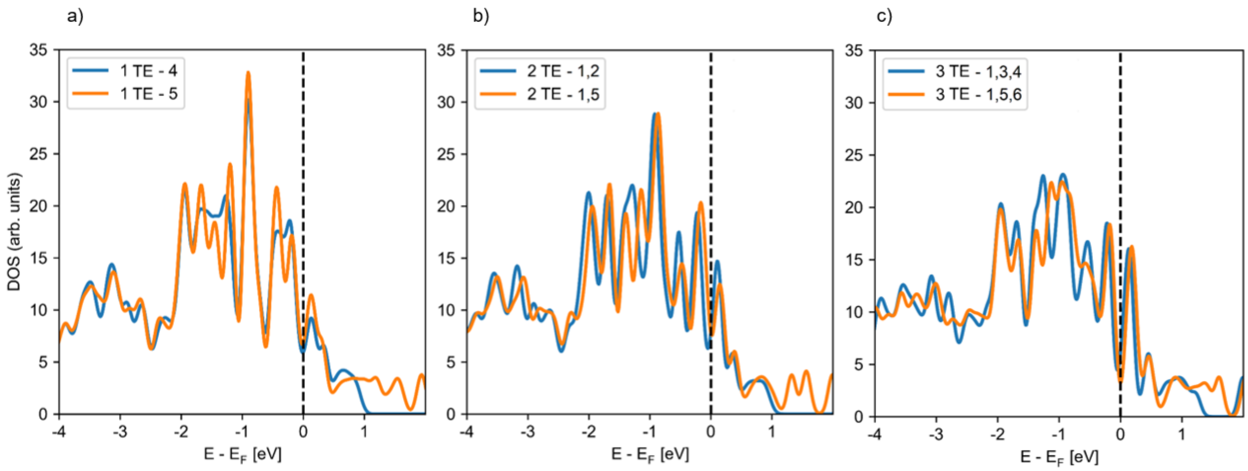

Figure S6: Comparison of total density of states of Pt_8 cluster for different positions of Ce^{3+} cations for 1 to 3 electrons transferred (a, b and c, respectively). The position of Ce^{3+} cations in the legends are depicted in Fig. 1.

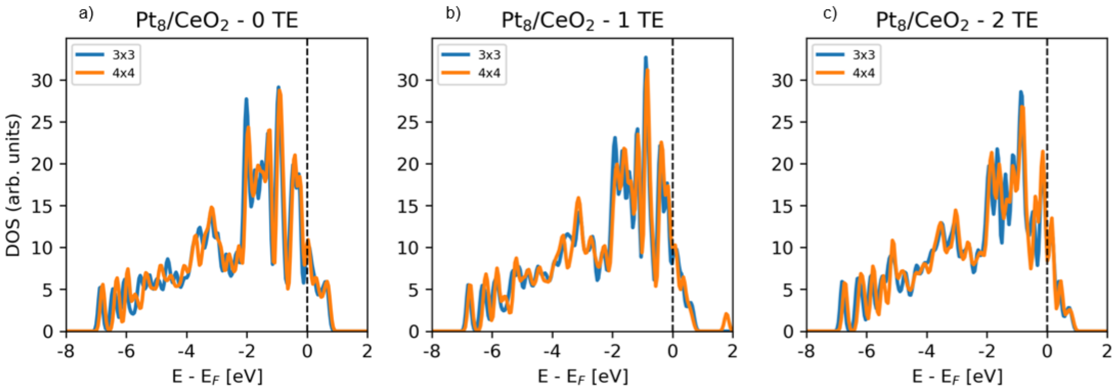

Figure S7: Comparison of total density of states of Pt_8 cluster for different supercell sizes for 0 to 2 transferred electrons (a, b and c, respectively). For 2 TE, the label 1,5 for the 4×4 supercell (Figure S1) was chosen because of its similar chemical environment to the state used for the supported Pt_8 on the 3x3 supercell (Figure 1). The most stable electronic states is evaluated in the 3×3 supercell (with Ce^{3+} in position Ce-5 for 1 TE and Ce-1/Ce-5 for 2 TE). The positions of Ce^{3+} cations in the 4×4 supercell were chosen to be the same with respect to the Pt_8 cluster as in the 3×3 supercell.

System	sp Integral	d Integral	Total Integral
0 TE	6.490	62.264	68.754
1 TE	6.459	62.201	68.66
2 TE	6.583	62.149	68.731
3 TE	6.603	62.043	68.646

Table S4: Integrated total, sp-orbitals and d-orbitals values of density of states (DOS) of the Pt_8 cluster at 0, 1, 2 and 3 transferred electrons.

Number of TE	no TE	1 TE		2 TE		3 TE	
Cluster atom	Ce4*f*-valence	Ce4*f*-core	Ce4*f*-valence	Ce4*f*-core	Ce4*f*-valence	Ce4*f*-core	Ce4*f*-valence
Pt-1	-0.14	-0.10	-0.11	-0.12	-0.12	-0.13	-0.13
Pt-2	-0.16	-0.14	-0.14	-0.13	-0.13	-0.11	-0.11
Pt-3	0.01	0.04	0.04	0.10	0.10	0.14	0.14
Pt-4	0.01	0.04	0.05	0.10	0.10	0.15	0.15
Pt-5	0.18	0.21	0.21	0.23	0.23	0.25	0.25
Pt-6	0.25	0.27	0.28	0.32	0.32	0.36	0.36
Pt-7	0.20	0.23	0.23	0.22	0.22	0.26	0.26
Pt-8	-0.10	-0.08	-0.07	-0.02	-0.02	0.11	0.11
Total Pt_8 Charge	0.25	0.47	0.49	0.70	0.70	1.03	1.03

Table S5: Bader charges of Pt atoms in the CeO_2(111)-supported Pt_8 cluster for 0 to 3 transferred electrons. The most stable electronic state is evaluated in each case, comparing values obtained with Ce4*f*-core and Ce4*f*-valence potentials. The cluster atoms are labelled as shown in Fig. 1.

Number of TE	1 TE		2 TE		3 TE	
Cluster atom	Ce-5	Ce-4	Ce-1,5	Ce-1,2	Ce-1,5,6	Ce-1,3,4
Pt-1	-0.11	-0.11	-0.12	-0.13	-0.13	-0.1
Pt-2	-0.14	-0.14	-0.13	-0.11	-0.11	-0.14
Pt-3	0.04	0.06	0.1	0.09	0.14	0.12
Pt-4	0.05	0.05	0.1	0.09	0.15	0.13
Pt-5	0.21	0.21	0.23	0.23	0.25	0.26
Pt-6	0.28	0.28	0.32	0.31	0.36	0.34
Pt-7	0.23	0.23	0.22	0.23	0.26	0.24
Pt-8	-0.07	-0.07	-0.02	-0.01	0.11	0.07
Total Pt_8 Charge	0.49	0.51	0.7	0.7	1.03	0.92

Table S6: Bader charges of Pt atoms in the CeO_2(111)-supported Pt_8 cluster, comparing different positions of Ce^{3+} cations for different number of transferred electrons. The numbers next to Ce indicate the position of formed Ce^{3+} cations and the Pt atoms labelled as indicated in Fig. 1.

Number of TE	0 TE		1 TE		2 TE	
Cluster Atom	3×3	4×4	3×3	4×4	3×3	4×4
Pt-1	-0.14	-0.15	-0.10	-0.13	-0.12	-0.13
Pt-2	-0.16	-0.12	-0.14	-0.12	-0.13	-0.11
Pt-3	0.01	-0.01	0.04	0.03	0.10	0.06
Pt-4	0.01	0.00	0.04	0.02	0.10	0.06
Pt-5	0.18	0.20	0.21	0.21	0.23	0.24
Pt-6	0.25	0.24	0.27	0.27	0.32	0.32
Pt-7	0.20	0.20	0.23	0.22	0.22	0.23
Pt-8	-0.10	-0.10	-0.08	-0.09	-0.02	0.02
Total Pt_8 Charge	0.25	0.26	0.47	0.42	0.70	0.69

Table S7: Bader charges of Pt atoms in the CeO_2(111)-supported Pt_8 cluster for 0 to 2 transferred electrons on the 4×4 CeO_2 slab. The most stable electronic state is evaluated in the 3×3 supercell, and the positions of Ce^{3+} cations in the 4×4 supercell were chosen to be the same with respect to the Pt_8 cluster as in the 3×3 supercell. All values were obtained using Ce4*f*-core potentials and spin-restricted calculations. The Pt atoms are labelled in Figure S1.

Adsorption site, theory level	0 TE	1 TE		2 TE		3 TE	
		Ce-5	Ce-4	Ce-1,5	Ce-1,2	Ce-1,5,6	Ce-1,3,4
Interface							
Pt-8, 4*f*-core-sr	-1.80	-1.91	-1.94	-2.09	-2.10	-2.34	-2.24
Pt-8, 4*f*-core-su	-1.78	-1.89		-2.12	-2.14	-2.37	-2.22
Pt-8, 4*f*-valence-su	-1.78			-2.02		-2.30	
Second-layer							
Pt-1, 4*f*-core-sr	-2.46	-2.40	-2.45	-2.36	-2.43	-2.35	-2.31
Pt-1, 4*f*-core-su	-2.43	-2.38	-2.42	-2.37	-2.44	-2.38	-2.30
Pt-1, 4*f*-valence-su	-2.43	-2.30		-2.42		-2.34	

Table S8: CO adsorption energies (in eV) on the interface Pt-8 and second-layer Pt-1 sites of Pt_8/CeO_2 for the electronic states with 0 to 3 transferred electrons (TE) from Pt_8, calculated at several levels of theory, including spin-restricted (sr) and spin-unrestricted (su) calculations using the 4*f*-core potential for describing Ce^{3+} cations, and spin-unrestricted calculations using the 4*f*-valence potentials only. Missing values indicate that those states could not be converged to at the corresponding level of theory.

CO Adsorption Energy [eV]		
	3×3	4×4
0 TE	-1.80	-1.75
1 TE	-1.91 (5)	-1.82 (5)
2 TE	-2.09 (1,5)	-2.20 (1,5)

Table S9: CO adsorption energies for different Pt_8 coverages corresponding to the 3×3 and 4×4 supercells. In parentheses are labels of positions of Ce^{3+} ions, see Figures 1 (3×3) and S1 (4×4). All adsorption energies are calculated at 4*f*core-sr level.

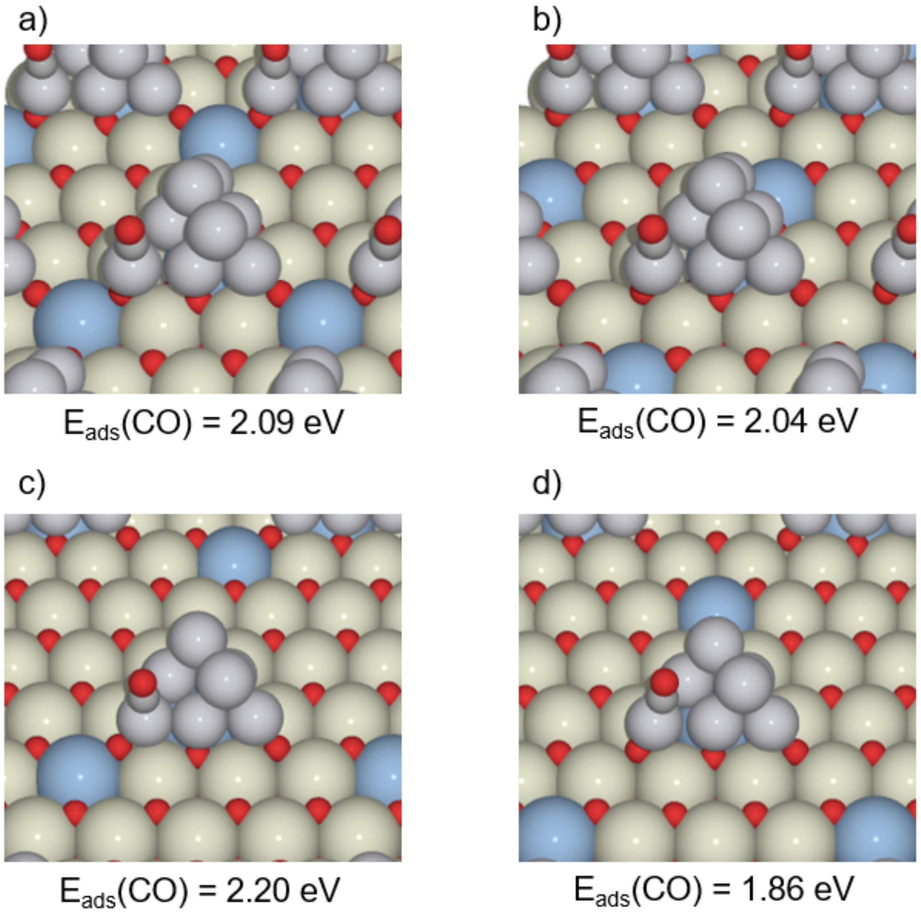

Figure S8: Optimized structures of the $CO/Pt_8/CeO_2(111)$ models, in which CO molecule is adsorbed on the interface site in the 3×3 and 4×4 supercells, and the corresponding CO adsorption energies. a) – label 1,5 and b) – label 2,5 (3×3, Figure 1); c) – label 1,5 and d) – label 5,7 (4×4, Figure S1). Color coding of atoms: Pt – light grey, Ce^{4+} – beige, Ce^{3+} - light blue, O – red, C – dark grey.

Adsorbate	H		H_2O		OH		CO	
Adsorption site	Inter-face	Second layer	Inter-face	Second layer	Inter-face	Second layer	Inter-face	Second layer
0 TE	-0.54	-0.79	-0.37	-0.75	-3.45	-4.03	-1.80	-2.46
1 TE	-0.60	-0.73	-0.44	-0.73	-3.48	-3.96	-1.91	-2.45
2 TE	-0.74	-0.70	-0.62	-0.71	-3.65	-3.96	-2.09	-2.43
3 TE	-0.87	-0.68	-0.85	-0.71	-3.76	-4.00	-2.34	-2.31

Table S10: Adsorption energies (in eV) of H, H_2O, OH, and CO adsorbates on the interface Pt-8 and the second-layer Pt-1 adsorptions sites, at 0 to 3 transferred electrons, calculated spin-restricted. The positions of Ce^{3+} cations described by the Ce4*f*-core potential are Ce-5 for 1 TE, Ce-1,5 for 2 TE, and Ce-1,5,6 for 3 TE.
